# People With Haemophilia as Data Coordinators: An Analysis of the Ethics and Feasibility of Self‐Management With Personal Health Records

**DOI:** 10.1111/hae.70120

**Published:** 2025-09-02

**Authors:** Martijn R. Brands, Lieke Baas, Mariette H. Driessens, Samantha C. Gouw, Rieke van der Graaf, Karina Meijer, for the SYMPHONY consortium

**Affiliations:** ^1^ Department of Pediatric Hematology Amsterdam UMC Location University of Amsterdam Amsterdam the Netherlands; ^2^ Department of Bioethics and Health Humanities Julius Center for Health Sciences and Primary Care University Medical Center Utrecht, Utrecht University Utrecht the Netherlands; ^3^ Netherlands Hemophilia Patient Society NVHP Nijkerk the Netherlands; ^4^ Department of Hematology University Medical Center Groningen University of Groningen Groningen the Netherlands

**Keywords:** decision making, ethical analysis, health information exchange, health records, personal autonomy, self‐management, shared

## Abstract

**Background:**

People with haemophilia perform various self‐management tasks, supported by multiple health apps. Personal health records will enable individuals to access and add health information from different institutions in a single digital tool, providing an integrated overview of data. Later, individuals will also be able to share their data with health care providers and relatives. This creates a new role for users: Coordinator of data exchange.

**Objective:**

To analyze if and how personal health records contribute to self‐management, with a particular emphasis on the role of coordinating data exchange.

**Methods:**

We applied various interpretations of self‐management to the promises of personal health records to identify what goals it intends to achieve. We then assessed various skills and responsibilities that are required from users to work with personal health records. Last, we analyzed potential scenarios of the coordination of data exchange.

**Results:**

Personal health records promise to support both compliant self‐management (i.e., managing care according to medical regimens) and concordant self‐management (i.e., managing care according to personal values and goals). Which of these forms is promoted depends on the goal of data coordinating tasks. The chosen design of the data sharing feature may impact the usability and accessibility of personal health records for a wide group of users.

**Conclusion:**

What form of self‐management is promoted by personal health records needs to be more clearly defined. A participatory design strategy can ensure that the design of coordinating data exchange matches individuals’ and health care providers’ needs.

## 1 Introduction

People with chronic and/or complex health conditions, such as haemophilia, often perform various self‐management tasks. People who are severely affected by haemophilia are at risk of experiencing spontaneous joint or muscle bleeding that may result in joint damage, for which lifelong monitoring and treatment are required. To prevent bleeds, regular self‐administered prophylactic infusions are necessary. In case of a bleed, individuals administer treatment according to their individual treatment plan, with remote advice provided by healthcare providers. Other self‐management tasks include: registering medication administrations in a digital journal [1], filling in questionnaires in an app, ordering medication, and checking laboratory results in a patient portal to monitor their condition.

To facilitate these self‐management tasks, individuals can make use of several health apps as well as patient portals, offered by most health care institutions in the Netherlands. As a result, individuals receiving care in multiple institutions must access different patient portals, with limited to no data exchange between them. The use of multiple apps and patient portals may result in an experienced lack of overview and control, which can be especially challenging for (older) people with haemophilia with comorbidities, who may have up to 15 different health care providers working in multiple care institutions [2]. Since it is often assumed that access to health information enables individuals to better monitor their condition(s) and treatment(s), an easy and comprehensive health overview is needed [3, 4, 5].

Personal health records combine information from multiple care providers in one website or app, thereby offering individuals a complete overview of their health information (see Box 1) [6, 7]. Although still under development, it is expected that one personal health record can replace the numerous patient portals and health apps currently utilized by health care recipients. Consequently, people with a chronic and/or complex condition such as haemophilia are often considered the primary beneficiaries of the personal health record [8].

**BOX 1 Definitions of patient portals and personal health records [4]**

- Patient portal: the patient‐facing interface of an electronic health record (the digital version of a health care provider's paper chart). Patient portals enable people to view (part of) their medical record, such as test results, medication or therapeutic instructions. Health care providers or institutions determine what information is accessible. Additional features are often present, including: messaging with health care providers, requesting prescription refills, appointment planning, filling in questionnaires or adding self‐measurements (e.g., body weight).
- Personal health record (PHR): PHRs generally have similar features as patient portals but differ in that contents are managed and maintained by individuals, not health care providers. People can access, manage and share their health information with other health care providers or relatives. Health information from different institutions can be accessed in one PHR. Globally, many PHRs are not yet linked to electronic health records, but all Dutch PHRs are.

**FIGURE 1:**
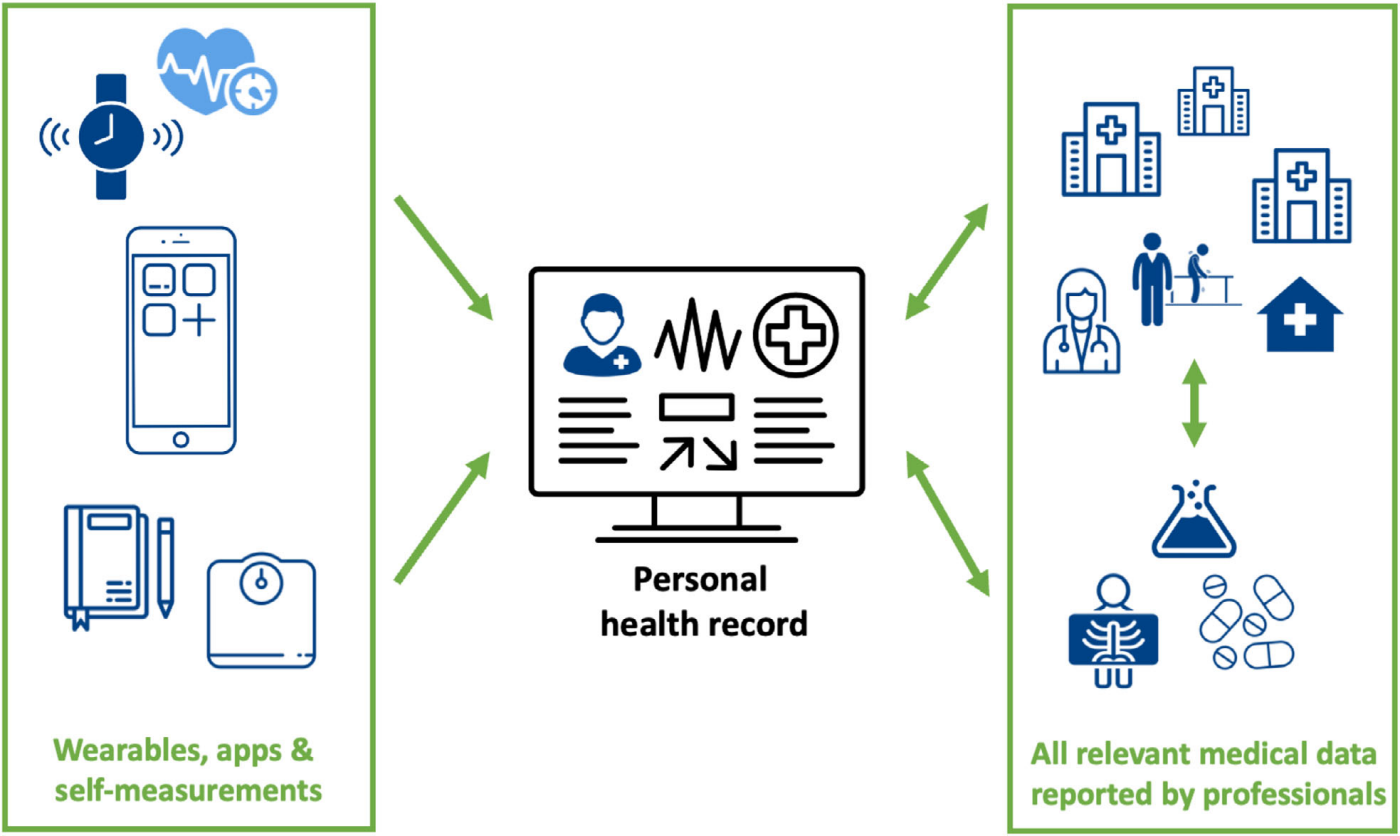
Schematic representation of the personal health record.

Personal health records are intended to offer three distinct features: viewing, adding and sharing health information [9]. First, personal health records promise individuals integrated access to all relevant health information from all healthcare institutions in one website or app, regardless of the care institution they are treated in, as illustrated in Figure 1 [6, 7]. Second, health care recipients can add information, including self‐measurements, questionnaire responses, a treatment diary or data from connected wearables. Individuals can either add data at their own initiative or the request of health care providers. Third, in the future, personal health records aim to enhance health care recipients’ control over their data by enabling them to share their digital data with family members, caretakers and health care providers [9]. In other words, personal health records can be considered personal data vaults, in which the individual controls access to all medical and self‐reported data. This coordination of data exchange, which requires determining what health information may be viewed by whom, is a new role for care recipients [10, 11, 12, 13]. This new role aims to increase the healthcare recipients’ level of self‐management. The goal of personal health records is the most contested amongst stakeholders [14].

In this paper, we identify several ethical and practical questions that arise when care recipients use a personal health record. In doing so, we intend to add to the debate on the responsible development of personal health records and, more specifically, their data‐sharing feature. While personal health records are being developed in many countries [15], few countries have formalized the development and implementation of personal health records as extensively as the Netherlands [16]. Therefore, we will focus on the situation in the Netherlands.

## 2 Methods

We first provide an overview of the discussions surrounding self‐management and apply these to the use of personal health records by people with haemophilia. Based on this, we raise the question of what goals self‐management through personal health records aims to achieve and how these goals may actually be supported by personal health records. We then discuss several skills that users of personal health records need to have and evaluate their impact on the accessibility of these tools. Last, we critically assess the different options under consideration for the design of the data sharing feature and end by suggesting an alternative variant.

## 3 Results and Discussion

### 3.1 Identifying the Goals of Self‐Management

Self‐management in health care is defined as ‘an individual's ability to detect and manage symptoms, treatment, physical and psychosocial consequences and lifestyle changes (such as exercise and diet) inherent in living with a chronic condition’. [17] Increasing self‐management in health care is considered to be desirable for several reasons, such as: more efficient disease management, leading to a decrease in health care costs; the contribution of self‐management to patient autonomy and independence; and health care recipients’ unique first‐person perspective to their body and life, which is essential to effective disease management [11, 18].

Self‐management can comprise many different activities, from administering prophylactic treatment to checking test results and uploading self‐measurements. Three forms of self‐management are distinguished in the literature [11]. In the first, individuals take over practical tasks and responsibilities from healthcare professionals. For people with haemophilia, this includes tasks such as administering prophylactic medication as prescribed and recording treatment and bleeds in a digital treatment journal. In this form of self‐management, individuals’ own views are not involved, nor is any decision‐making required from them [11].

In the second form, referred to as compliant self‐management, individuals learn to manage the disease or condition on their own. This includes learning to interpret bleeding symptoms and health data and knowing how to act in response. This form of self‐management increases individuals’ independence from their health care provider but does so in a way that mainly promotes compliance with medical regimes [11]. Many of the telehealth interventions currently developed for the self‐management of haemophilia aim to promote such adherence to treatment plans [19].

The third form of self‐management, referred to as concordant self‐management, describes a form of self‐management in which individuals are enabled to find their way of living with the condition according to their personal views, values and goals [11]. Importantly, these do not have to overlap with what is optimal from a medical perspective [11].

It has been argued that successful self‐management can be achieved through empowerment [17]. There is no consensus, however, on how empowerment should be conceptualized and what it entails in relation to digital health tools [20, 21, 22, 23, 24]. Although there are ambiguities, patient empowerment is generally considered to be desirable and understood to be a process or a state wherein health recipients have or obtain the ability to take control over their health and well‐being, rather than ceding such control to a healthcare provider [22, 24, 25]. It is left unspecified exactly how this control is gained [21]. In the literature on digital health tools, it is often presumed that obtaining more information will lead to more control [13, 21]. Different authors describe various attributes that are required to obtain this control, such as having sufficient knowledge, skills and self‐determination [22, 23, 25]. Many digital health tools, including personal health records, have as one of their most important aims that they provide health care recipients with information. However, increasing the amount of information is not sufficient for increasing care recipients’ knowledge or their ability to make and execute choices regarding their health [13, 21]. Furthermore, it is generally argued that obtaining control over health requires more than control over circumstances and the ability to make choices [21, 23]. Therefore, some authors argue that digital health tools may primarily give users the feeling of being empowered, while empowering effects are often disproportionate to the burden of additional responsibilities assigned to users [13].

It is generally assumed that personal health records will increase care recipients’ self‐management and support them to become empowered. It is said that through easier, integrated access to health information, personal health records may increase health knowledge and disease insight, [8, 14, 26, 27] help people to better consider their own preferences, wishes and values, facilitate disease management [8, 14, 26] and enable individuals to take on a more pro‐active role in and increased control over their health care [26].

Based on the literature on personal health records, it appears personal health records aim to support both compliant and concordant forms of self‐management, by assuming individuals will adopt a healthier lifestyle and simultaneously supporting them to live a life according to their own values and goals. However, these aims can easily compete with each other, since pursuing one's own goals does not necessarily imply pursuing goals that align with medical advice. For instance, people with haemophilia might aspire to a career as a police officer or desire to play a game of rugby. Making other decisions than those prescribed by health care providers or an individual's refusal to take on certain tasks or responsibilities can also be an expression of their autonomy and a sign of empowerment. [12, 17]

Furthermore, it is questionable if control over data sharing aims to support the same goals and values as control over health does. Control over data sharing is not as directly related to health outcomes or the ability to live a life according to one's views, which are some goals of increasing self‐management in health care. Instead, control over data coordination aims to increase privacy and insight into the availability of data [28].

There should thus be a more explicit recognition of the exact goals of self‐management and how the personal health record aims to support these. Crucially, supporting concordant self‐management also means that people with haemophilia should have the opportunity to refrain from using a personal health record if they want to.

### 3.2 Skills Required for Becoming a ‘Data Coordinator’

Data coordination requires several skills and knowledge to successfully lead to empowerment. In particular, four forms of literacy can be distinguished that are relevant to the use of personal health records [29]. First, general literacy refers to the ability to read written text and to understand the language it is written in, as well as numeracy skills [30].

Second, computer and mobile device literacy refers to skills needed to access the internet, use a computer or mobile device or perform online authorization for government and health services [31]. To exercise computer skills, people need to have sufficient fine motor skills (needed to use a mouse, keyboard or phone) and have no clinically relevant visual impairments [30].

Third, health literacy is defined as ‘people's knowledge, motivation and competences to access, understand, appraise and apply health information in order to make judgements and take decisions in everyday life concerning healthcare, disease prevention and health promotion’. [32] In the coordination of data exchange, this also requires an understanding of the demarcation of medical specialities (i.e., what parts of the human body does a urologist treat) and which professionals collaborate in shared care and what information is relevant for health care providers’ medical reasoning process. Fourth, information literacy refers to people knowing how information is organized and how to find information [33].

Due to these essential skills, as well as the required effort, responsibilities and understanding, the use of a personal health record may be less accessible to some people than to others [12]. This may exclude certain groups. This is a risk that arises with many digital health tools [10].

### 3.3 Two Scenarios for Data Exchange Coordination Through the Personal Health Record

In addition to integrating digital self‐managing tasks, personal health records also aim to increase self‐management in a new way by enabling the coordination of data exchange. As a result, individuals will then become ‘data coordinators’ of their health care data [26]. No personal health records currently enable this.

Currently, health care recipients can view their medical records digitally and have the right to request removal or correction of health information in medical records, which health care providers approve on a case‐by‐case basis [34]. However, care recipients are not able to electronically share their data with health care providers proactively. Consequently, health care providers can only directly access health information reported by their colleagues in three situations. First, if colleagues work in the same care institution and therefore use the same electronic health record. Second, if there is presumed consent of individuals (e.g., an individual agrees to be referred to another health care provider). Third, if two health care providers work together in treating the same condition (e.g., shared care) [35, 36]. In all other cases, care recipients must first approve data exchange. As a result, the required information can often only be accessed after a first consultation, although access to health information is essential for health care providers’ medical reasoning and (shared) decision making.

For the design of data sharing in the personal health record, two potential scenarios are considered by legislative parties, each with its advantages and limitations [37]. In the ‘active’ design scenario, individuals are given the option to proactively share health data with health care providers. Individuals may determine the recipient of health information (e.g., general practitioners, hospitals, mental health facilities), what information is shared (e.g., medication, treatment plans) or both [38]. In this ‘active’ scenario, prior to a consultation with a health care provider, health care recipients need to independently determine which information to share and not to share, by assessing what information would be of interest to a particular health care provider.

In contrast to the ‘active’ design scenario, in the ‘passive’ scenario, a health care provider asks individuals to share (part of) their health data prior to a consultation [37]. Instead of leaving it to care recipients to assume what information is important to a certain health care provider, it would be the health care provider's responsibility to determine and communicate which information is relevant to them. Individuals would then only decide to share or not share the requested information. Yet, this would require health care providers to determine, well in advance of a consultation, what information might be relevant to them and would require individuals to respond to these requests. It is uncertain if this is feasible for health care providers.

Instead of each of these designs, a hybrid scenario, combining the two designs, might work best. In a hybrid design, an active scenario for general health information may be combined with a passive scenario for disease‐specific information. For general health information, such as medical history, medication prescriptions and medication administrations, healthcare users would be asked to proactively determine the recipient (e.g., general practitioner, hospitals). Consequently, individuals would only receive passive requests from health care providers to share disease‐specific information (e.g., a haematologist has asked a person with haemophilia to record bleeds in a digital treatment diary). Individuals would still be able to refrain from sharing health information after discussing this with health care providers on a case‐by‐case basis [31].

Nonetheless, several challenges associated with the role of data coordinator will remain in a hybrid design, such as those related to health recipients’ skills, literacy and the transfer of responsibilities. Counselling individuals on data sharing decisions may help to strengthen their self‐efficacy (i.e., the belief to be able to successfully execute self‐management tasks) [39], resulting in less hesitancy over data sharing choices and reduced stress [40].

## 4 Conclusion

By offering individuals easier, integrated access to health information and more control over the exchange of this data, personal health records aim to increase individuals’ self‐management. This may especially benefit individuals with chronic and/or complex health conditions, such as haemophilia. We evaluated if and how personal health records can achieve this goal by distinguishing between compliant and concordant self‐management. Which of the two forms is primarily supported partly depends on the goals of increasing self‐management. These may include, but are not limited to, increasing efficiency in health care, supporting patient autonomy and increasing privacy. The goal needs to be better defined before data sharing through the personal health record can truly aid empowerment. Furthermore, there may be differences between the skills and wishes of various health care recipients or parents/caregivers of health care recipients, which need to be accommodated. Therefore, we recommend a participatory design strategy to determine the best design scenario for data sharing in the personal health record and to ensure individuals receive sufficient support to make decisions on data sharing.

A participatory approach to the governance of personal health records remains necessary once they are implemented, as their use may change over time. For instance, although personal health records currently do not yet aid secondary use of data, Dutch government officials have already hinted at this future iteration. Moreover, the European Health Data Space (EHDS), which aims to establish a European Union‐wide digital infrastructure for individuals to share their health data, stresses the importance of the secondary use of health data for, amongst others, research. Personal health records make up a critical part of the infrastructure that the EHDS envisions. Continuous involvement of all stakeholders in the governance of personal health records can help ensure that such developments align with the needs of stakeholders.

## Author Contributions

Martijn R. Brands and Lieke Baas drafted the manuscript and contributed equally. All authors provided feedback and approved the final manuscript.

## Ethics Statement

No research with humans or animals was conducted for this study. Ethics approval is not required.

## Conflicts of Interest

S.C. Gouw received an unrestricted medical research grant from Sobi. K. Meijer reports speaker fees from Alexion, participation in trial steering committees for Bayer and Astra Zeneca, consulting fees from Therini, participation in data monitoring and endpoint adjudication committee for Octapharma.

## Data Availability

Data sharing is not applicable to this article as no datasets were generated or analysed during the current study.

## References

[1] HemoNED Foundation , “VastePrik Digital treatment diary,” (HemoNED Foundation, 2023).

[2] C. Smit , “Cees Smith Testimonial,” (Chrodis, 2015).

[3] H. R. Han , K. T. Gleason , C. A. Sun , et al., “Using Patient Portals to Improve Patient Outcomes: Systematic Review,” JMIR Human Factors 6, no. 4 (2019): e15038. 31855187 10.2196/15038 PMC6940868

[4] M. R. Brands , S. C. Gouw , M. Beestrum , R. M. Cronin , K. Fijnvandraat , and S. M. Badawy , “Patient‐Centered Digital Health Records and Their Effects on Health Outcomes: A Systematic Review,” Journal of Medical Internet Research 24, no. 12 (2022): e43086. 36548034 10.2196/43086 PMC9816956

[5] “Individuals' Right Under HIPAA to Access Their Health Information 45 CFR § 164.524” (U. S. Department of Health and Human Services, 2024). Accessed Mar 1, 2024. https://www.hhs.gov/hipaa/for‐professionals/privacy/guidance/access/index.html.

[6] “Stand van Zaken Persoonlijke Gezondheidsomgevingen,” Het Ministerie van Volksgezondheid, Welzijn en Sport, 2022.

[7] “Voortgang Persoonlijke Gezondheidsomgevingen,” Het Ministerie van Volksgezondheid,Welzijn en Sport, 2023.

[8] I. Vajda , J. Goedhoop , and E. van Gelder , “Doorontwikkeling van een PGO: Van de vraag van gebruikers naar bruikbare functies,” (PGO alliantie, 2021).

[9] M. Brands , S. Gouw , and M. Driessens , “Personal Health Records: A Promising Tool? (Persoonlijke gezondheidsomgeving: Een veelbelovend hulpmiddel?),” Nederlands Tijdschrift Voor Geneeskunde 16, no. 167 (2023): D6908. 36928474

[10] F. Lucivero and K. R. Jongsma , “A Mobile Revolution for Healthcare? Setting the Agenda for Bioethics Political” Journal of Medical Ethics 44 (2018): 685–689. 29907579 10.1136/medethics-2017-104741 PMC6173811

[11] M. Schermer , “Telecare and Self‐Management: Opportunity to Change the Paradigm?,” Journal of Medical Ethics 35 (2009): 688–691. 19880706 10.1136/jme.2009.030973

[12] B. Prainsack , Personalized Medicine: Empowered Patients in the 21st Century? (NYU Press, 2017): 1–288.

[13] J. Gray , S. Segers , and H. Mertes , “The Information, Control, and Value Models of Mobile Health‐Driven Empowerment,” Bioethics 10, no. January (2024): 1–7. 10.1111/bioe.13294 38662961

[14] R. van der Ploeg , K. Waaijer , S. Stam , and B. Pluut , “Herijkte Visie op Persoonlijke GezondheidsOmgevingen (PGO),” (Pluutpartners, 2023), https://pluutpartners.nl/wp‐content/uploads/2023/05/Een‐herijkte‐visie‐op‐de‐PGO.pdf.

[15] “Exchange of Electronic Health Records Across the EU,” (European Union, 2022), https://digital‐strategy.ec.europa.eu/en/policies/electronic‐health‐records.

[16] “Wegiz: Wet Elektronische Gegevensuitwisseling in De zorg,” (Nictiz, 2023), https://nictiz.nl/wat‐we‐doen/programmas/werken‐aan‐wegiz/.

[17] B. K. Redman , “Responsibility for Control: Ethics of Patient Preparation for Self‐Management of Chronic Disease,” Bioethics 21, no. 5 (2007): 243–250. 17845469 10.1111/j.1467-8519.2007.00550.x

[18] S. Holm , “Justifying Patient Self‐Management—Evidence Based Medicine or the Primacy of the First Person Perspective,” Medicine, Health Care, and Philosophy 8, no. 2 (2005): 159–164. 16215795 10.1007/s11019-005-2280-x

[19] W. Qian , T. T. N. Lam , H. H. W. Lam , C. K. Li , and Y. T. Cheung , “Telehealth Interventions for Improving Self‐Management in Patients With Hemophilia: Scoping Review of Clinical Studies,” Journal of Medical Internet Research 21 (2019): e12340. 31293241 10.2196/12340 PMC6652120

[20] T. Risling , J. Martinez , J. Young , and N. Thorp‐Froslie , “Evaluating Patient Empowerment in Association With ehealth Technology: Scoping Review,” Journal of Medical Internet Research 19 (2017): e329. 28963090 10.2196/jmir.7809 PMC5640823

[21] A. Kapeller and I. Loosman , “Empowerment Through Health Self‐Testing Apps? Revisiting Empowerment as a Process,” Medicine, Health Care, and Philosophy 26, no. 1 (2023): 143–152. 36592301 10.1007/s11019-022-10132-w PMC9806806

[22] P. Bravo , A. Edwards , P. J. Barr , I. Scholl , G. Elwyn , and M. McAllister , “Conceptualising Patient Empowerment: A Mixed Methods Study,” BMC Health Services Research 15, no. 1 (2015): 1–14. 26126998 10.1186/s12913-015-0907-z PMC4488113

[23] A. Kapeller , “Phenomenology and Empowerment in Self‐Testing Apps,” Bioethics 38 (2024): 770–777. 38639089 10.1111/bioe.13293

[24] “Health Promotion Glossary of Terms 2021,” (World Health Organization, 2021).

[25] K. V. Kreitmair , “Mobile Health Technology and Empowerment,” Bioethics 38 (2024): 481–490. 36950727 10.1111/bioe.13157

[26] Magazine Deel , “De patiënt Als CEO van Zijn Eigen Gezondheid: Een interview met de voorzitter van de patiëntenfederatie en de Secretaris Generaal van het Ministerie van Volksgezondheid,” Welzijn En Sport 2023, https://www.medmij.nl/wp‐content/uploads/2019/07/Magazine‐Deel‐Regie‐op‐Gegevens‐MedMij.pdf.

[27] M. G. H. Niezen and P. Verhoef , “Digitale Gezondheidsregie: Meer Gegevens, Meer Grip?,” (Rathenau Institute, 2018), https://www.rathenau.nl/sites/default/files/2018‐06/Rapport/Digitale/gezondheidsregie.pdf.

[28] J. Van den Hoven ,“Information Technology, Privacy, and the Protection of Personal Data,” in Information Technology and Moral Philosophy, ed. J. Van den Hoven , and J. Weckert (Cambridge University Press, 2008).

[29] R. Van Der Vaart and C. Drossaert , “Development of the Digital Health Literacy Instrument: Measuring a Broad Spectrum of Health 1.0 and Health 2.0 Skills,” Journal of Medical Internet Research 19, no. 1 (2017): 1–13. 10.2196/jmir.6709 PMC5358017 28119275

[30] S. J. Czaja , C. Zarcadoolas , W. L. Vaughon , C. C. Lee , M. L. Rockoff , and J. Levy , “The Usability of Electronic Personal Health Record Systems for an Underserved Adult Population,” Human Factors 57, no. 3 (2015): 491–506. 25875437 10.1177/0018720814549238 PMC4710573

[31] A. Barakat , R. D. Woolrych , A. Sixsmith , W. D. Kearns , and H. S. Kort , “eHealth Technology Competencies for Health Professionals Working in Home Care to Support Older Adults to Age in Place: Outcomes of a Two‐Day Collaborative Workshop,” Med 2 0 , no. 2 (2013): e10. 25075233 10.2196/med20.2711 PMC4084768

[32] K. Sørensen , S. Van Den Broucke , J. Fullam , et al., “Health Literacy and Public Health: A Systematic Review and Integration of Definitions and Models,” BMC Public Health 12, no. 1 (2012): 80. 22276600 10.1186/1471-2458-12-80 PMC3292515

[33] C. D. Norman and H. A. Skinner , “eHealth Literacy: Essential Skills for Consumer Health in a Networked World,” Journal of Medical Internet Research 8, no. 2 (2006): 1–10. 10.2196/jmir.8.2.e9 PMC1550701 16867972

[34] “‘Rights Regarding Patients’ Medical Records” (Autoriteit Persoonsgegevens, 2024).

[35] “Medical Treatment Contracts Act (Wet op de geneeskundige behandelingsovereenkomst, WGBO),” (GDPR Helpdesk (AVG Helpdesk); Dutch Ministry of Health, 2024).

[36] “Toestemmingen: Toestemming voor het uitwisselen van medische gegevens tussen zorgverleners,” (Dutch Ministry of Health, 2021).

[37] Personal communication between Martijn Brands and MedMij relationship manager , 2024, www.medmij.nl.

[38] “Mitz,” (Vereniging van Zorgaanbieders voor Zorgcommunicatie (VZVZ), 2024).

[39] D. Schulman‐Green , S. Jaser , F. Martin , et al., “Processes of Self‐Management in Chronic Illness,” Journal of Nursing Scholarship 44, no. 2 (2012): 136–144. 22551013 10.1111/j.1547-5069.2012.01444.x PMC3366425

[40] K. Tahmassian and N. J. Moghadam , “Relationship Between Self‐Efficacy and Symptoms of Anxiety, Depression, Worry and Social Avoidance in a Normal Sample of Students,” Iranian Journal of Psychiatry and Behavioral Sciences 5, no. 2 (2011): 91–98. 24644452 PMC3939966

